# Burden of mental, behavioral, and neurodevelopmental disorders in the Finnish most preterm children: a national register study

**DOI:** 10.1007/s00787-023-02172-1

**Published:** 2023-02-27

**Authors:** Marika Leppänen, Bernd Pape, Liisi Ripatti, Max Karukivi, Leena Haataja, Päivi Rautava

**Affiliations:** 1 Neuropsychiatric Outpatient Clinic, Turku University Hospital, and Preventive Medicine, University of Turku, 20014 Turun Yliopisto, Turku, Finland; 2 Department of Mathematics and Statistics, University of Vaasa, and Turku University Hospital, Turku, Finland; 3 Department of Pediatric Surgery, Turku University Hospital, Turku, Finland; 4 Department of Adolescent Psychiatry, University of Turku, and Turku University Hospital, Turku, Finland; 5 Department of Pediatric Neurology, Pediatric Research Centre, University of Helsinki, and Children’s Hospital, Helsinki University Hospital, Helsinki, Finland; 6 Research Services, Turku University Hospital, and Preventive Medicine, University of Turku, Turku, Finland

**Keywords:** Prematurity, Maternal factors, Development, Mental health

## Abstract

Etiologies and the whole picture in childhood mental, behavioral, and neurodevelopmental disorders related to gestational age are unclear. This study included all Finnish children (*N* = 341,632) born between January 1, 2001, and December 31, 2006, whose data including their mothers (*N* = 241,284) were collected from national registers. Children with unclear gestational age (GA) (*N* = 1245), severe congenital malformations (*N* = 11,746), and moderate/severe/undefined cognitive impairment (*N* = 1140), and those who died during the perinatal period (*N* = 599) were excluded. The main outcome was the prevalence of mental and behavioral disorders (International Classification of Disorders) at 0 − 12 years of age in association with GA, adjusted for gender and prenatal variables. Out of all included (*N* = 326,902) children 16.6% (*N* = 54,270) were diagnosed to have any mental health disorder at 0 − 12 years. Adjusted Odd Ratio (OR) were for any disorder in preterm (< 37 weeks) 1.37 [1.28 − 1.46] and 4.03 [3.08 − 5.26] in extreme preterm (≤ 28 weeks) versus term born children, *p* < 0.05. The lower the GA at birth, the higher the risk for multiple disorders and earlier onset of disorder, *p* < 0.05. Adjusted ORs were for male/female 1.94 [1.90 − 1.99], maternal mental health disorder (yes/not) 1.99 [1.92 − 2.07], and smoking during pregnancy (yes/not) 1.58 [1.54 − 1.62], and these risks were more common in preterm versus term born children (*p* < 0.05). Extreme early birth was a strong risk factor per se for any or multiple and early shown mental health disorders. Other risk factors for mental health accumulated to preterm children.

**Supplementary Information:**

The online version contains supplementary material available at 10.1007/s00787-023-02172-1.

## Introduction

Earlier studies [1–10] have replicated the finding that preterm (PT, < 37 weeks of gestation) birth, especially extreme prematurity (≤ 28 weeks), is a risk by itself for mental health disorders. Neurodevelopment of a former PT child can be delayed in one or in multiple domains [1, 4, 11–13]. Also likelihood of a specific disorder [3, 9, 14–16] seems to increase by decreasing gestational age (GA). Multiple pre- and postnatal risk factors have been identified [4, 7, 17–19].

When considering the entire family system, the major strain and stress [20, 21] for parents related to prematurity is usually during the early years [22]. This may be one of the reasons why PT children under 5 years are found to be at risk of being removed from the home [23]. Regarding mental and neurodevelopmental outcomes, it is essential to acknowledge that caregivers have the potential to attenuate [24, 25] or to step-up [18] the biological vulnerability of their child. Thus, factors that may affect negatively on parenting [26], and especially in mothers [27, 28], such as their young age or low education level [9], and mental health disorders [10], may relate to the likelihood of mental health problems in offspring [29]. Furthermore, PT birth has been shown to be more common in mothers with mental health problems [30, 31] and in poor socioeconomic situation [31, 32], which may increase possible inequality [33] and the need for psychosocial support after PT birth [3, 34, 35].

Earlier register studies assessing mental health outcomes in former PT infants have focused on late outcomes in certain disorder at one age without longitudinal follow-up of mental health disorders [3, 8, 9, 36]. Though, deficits in socio-emotional or learning functions [17], and mental health disorders can be diagnosed in infancy [7, 12, 18], or between pre-school and school age [4, 7, 37, 38]. In infants, emotional and behavioral problems may manifest, and be recognized, as dysregulation of age-typical behavior, like in crying, eating, and activity [7, 18, 19, 39]. The dysregulation at age of 10 months have been shown to predict neurodevelopmental disorders at age 5 − 7 years in PT born children, but probably not emotional disorders [18]. Further, also externalizing disorders, seem to be quite stable from early childhood to later childhood [1] and adolescence in PT children [10, 16, 40, 41]. This seems to be the pathway for many mental health disorder [42].

However, it would be important to find early symptoms as these may predict comorbidity in mental health disorders [14], adverse daily life functioning in childhood [4, 18, 43] and in adulthood [10, 40, 41]. In addition, it is still unclear what is the total burden of mental health disorders in childhood after PT birth [19, 40, 44] and if there are certain pathways in mental health disorder for different GA groups. There is also a lack of detailed knowledge of etiologies for different disabilities in this heterogenic PT group [9, 45–47].

The aim of this study is first to present the overall mental health morbidity pathway from birth to the age of 12 years in all Finnish children born in 2001 − 2006 with our register study. Further, the aim was to study how GA associates with mental health outcomes in childhood, and in what proportion the gender of the child and the health and life circumstances of the mother may predict the child’s mental health outcome. It was hypothesized that mental, behavioral, and neurodevelopmental disorders are more common in PT than in term children, and that other vulnerabilities in families may increase the risk of disorders.

## Methods

The Strengthening the Reporting of Observational Studies in Epidemiology (STROBE) cohort study checklist [48] was used as guidance for reporting this study. The pseudonymized data sources were linked together by the National Institute of Health and Welfare (THL). The study protocol was approved by the THL (THL/595/5.05.00/2019) and Turku University Hospital (J44/19). This was a retrospective register study, and no informed consent was required. The participants were not contacted. The legal basis for processing personal data is public interest and scientific research (EU General Data Protection Regulation 2016/679 (GDPR), Article 6(1)(e) and Article 9(2)(j); Data Protection Act, Sects. 4 and 6).

This register study is based on different THL registers, which were linked to each other to complete information on mental health disorders of mothers before pregnancy, perinatal data, and mental health disorders of children from birth until 12 years of age. The baseline characteristics were collected from the Finnish Medical Birth Register (MBR) [49], that contains information on the mother’s health and socioeconomic data during pregnancy, about delivery, and the newborn infant on all live births and stillbirths in Finland from GA of 22 + 0 weeks or with a birth weight of at least 500 g. In Finland, GA is determined by routine practice first-trimester and controlled in later ultrasounds. The Finnish Hospital Discharge Register (HDR) has records for all public inpatient and outpatient care periods, covering over 95% of special healthcare in- and outpatient visits in Finland [50]. Register on Congenital Malformations maintains a register of congenital chromosomal and structural anomalies. The International Classification of Diseases [51] has been used to code mental health-related disorders and diseases in Finland, currently 10th revision (ICD-10) is in use. In this manuscript, mental, behavioral, and neurodevelopmental disorders or mental health disorders are used synonymously.

All Finnish children (*N* = 341,632) born between January 1, 2001, and December 31, 2006, were included. We excluded perinatal deaths (stillborn and died about 7 days after birth, *N* = 599, 0.2%) and children with unclear GA (*N* = 1245, 0.4%). Further, major congenital malformations (*N* = 11,746, 3.4%) and severe, profound, or unspecified cognitive impairments by ICD-10 codes F72 − 73 and F79 (*N* = 1140, 0.3%) were left out to exclude their potential confounding effect for mental health disorder diagnostic.

Children were considered to have had a mental health disorder if they were diagnosed with any mental or behavioral disorder (the chapter F00 − 99 in ICD-10) during their first 12 years of life. In the same way, mothers were considered to have had a mental, behavioral, or neurodevelopmental disorder, if they had been diagnosed before the calendar year of their childbirth. This data was used to describe the mother’s mental health situation before pregnancy and birth of child.

GA was analyzed as dichotomized variable (term/preterm) and with preterm status further subdivided into the three stabilized groups by GA: extremely low GA (ELGA, ≤ 28 weeks), very low GA (VLGA, 29 − 31 weeks) and low GA (LGA, 32 − 36 weeks). Prior births, multiple birth, gender of the child, mother’s self-reported information on smoking, whether the mother was living alone or with a partner, and working status of mother when expecting child were categorized dichotomously as yes or no. The mother was classified as being out of work life if she had reported being unemployed, student, retired, or had reported to have family leave during pregnancy instead of being in working life.

The association between mental health morbidity and GA was assessed using logistic regressions, both with and without adjusting for the perinatal and socioeconomic factors mentioned above. We also examined the interactions between GA and those added factors, of which only those with previous pregnancies and multiple births turned out to be significant. A one-way ANOVA was used to assess differences in continuous variables, and chi-square tests were used to assess differences in categorical variables across the GA groups. All statistical tests were performed as 2-sided, with significance level set at 0.05. The analyses were performed using the SAS System, version 9.4 for Windows (SAS Institute Inc., Cary, NC, USA).

## Results

The final data consisted of 326,902 children, followed up until 12 years with their 241,284 mothers. Out of the included children, 94.7% were born at term, and 5.3% were PT. Of the PT children, 0.2% were born as ELGA, 0.7% as VLGA, and 4.3% as LGA. The baseline data of all children and their mothers are described in Table 1. All background characteristics differed between PT and term children (*p* < 0.001), except work status of mother (*p* > 0.05). Negative health and life circumstances of mothers were more common in PT when compared to children born at term.

**Table 1:** Background characteristics of the studied children and their mothers

Percentage (%) or mean (SD)	Preterm children *N* = 17,203				Term children *N* = 309,699
	All	ELGA (*N* = 796)	VLGA (*N* = 2229)	LGA (*N* = 14,178)	
Children					
Birth weight (grams)	2409 (682)	924 (286)	1621 (376)	2617 (522)	3587 (480)
Gender (female/male)	46.1/53.9	48.1/51.9	44.2/55.8	46.3/53.7	49.2/50.8
Mothers					
Prenatal mental health morbidity	7.8	8.0	8.8	7.7	6.5
Smoking during pregnancy	15.7	16.5	16.4	15.6	14.7
Age during labor (years)	29.9 (5.7)	30.6 (6.1)	30.3 (6.0)	29.8 (5.6)	29.4 (5.5)
Prior births (≥ 1)	47.9	47.1	44.1	48.5	58.5
Out-of-work life	16.4	16.7	16.4	16.4	16.8
Living in relationships	85.9	82.0	86.4	86.0	88.2
Multiple birth (≥ 1)	24.4	22.7	31.5	23.4	1.7

Extreme low GA (ELGA, ≤ 28 weeks), very low GA (VLGA, 29 − 31), low GA (LGA, 32 − 36) and term (≥ 37) children

Out of all included children, 16.6% (*N* = 54,270) were diagnosed to have at least one mental, behavioral, or neurodevelopmental disorder between birth and 12 years of age, 16.3% (*N* = 50,448) of term and 22.2% (*N* = 3822) of PT children, *p* < 0.0001. The prevalence in these disorders was highest for children born ELGA (42.6%), then for VLGA (28.3%), and LGA (20.1%), *p* < 0.0001. Out of all mental, behavioral, and neurodevelopmental disorder diagnoses, 44,091 were collected from specialized health care and/or 22,565 from the primary healthcare register. The distribution of mental, behavioral, and neurodevelopmental disorder diagnosis groups in all children is presented in the online resource, Table 1. The most common subchapter group, both in all (9.7%, *N* = 31,719) and in PT children (12.6%, *N* = 2167), was “Behavioral and emotional disorders usually occurring in childhood and adolescence” (F90 − 98). This contains a wide spectrum of different disorders, such as attention deficit hyperactive, conduct, emotional, tics and social disorders. The next most common disorder group both for all (7.3%, *N* = 23,801) and PT children (11.8%, *N* = 2035) was “Pervasive and specific developmental disorders” (F80 − 89), containing specific learning/scholastic/motor disorders and more profound disorders like autism spectrum disorder.

**Fig. 1:**
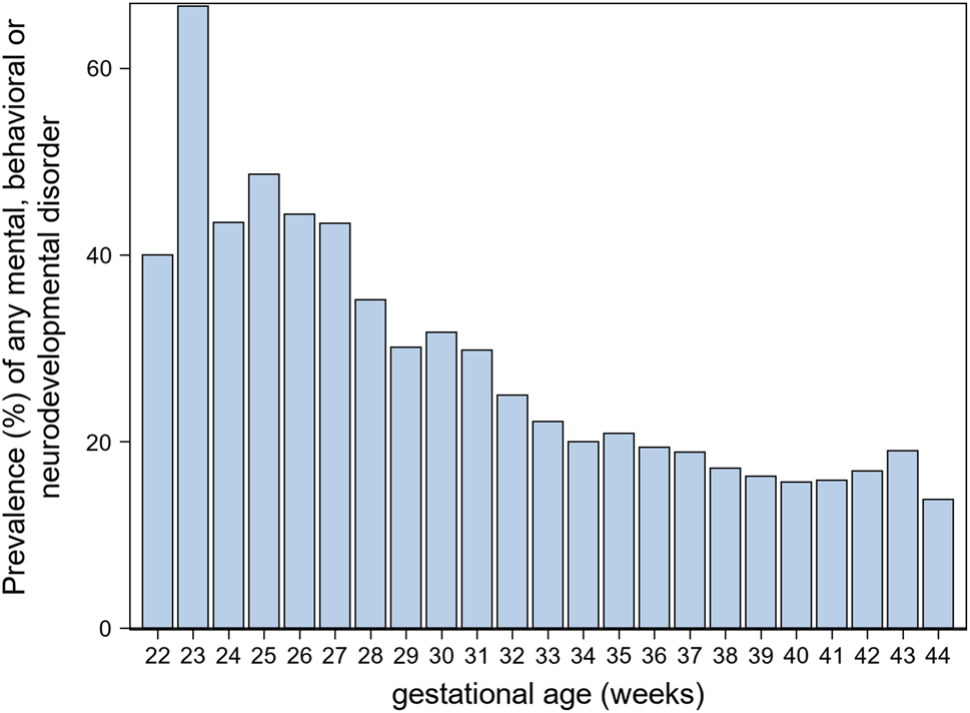
Prevalence of any mental, behavioral, or neurodevelopmental disorders during the first 12 years of life according to gestational age

**Fig. 2:**
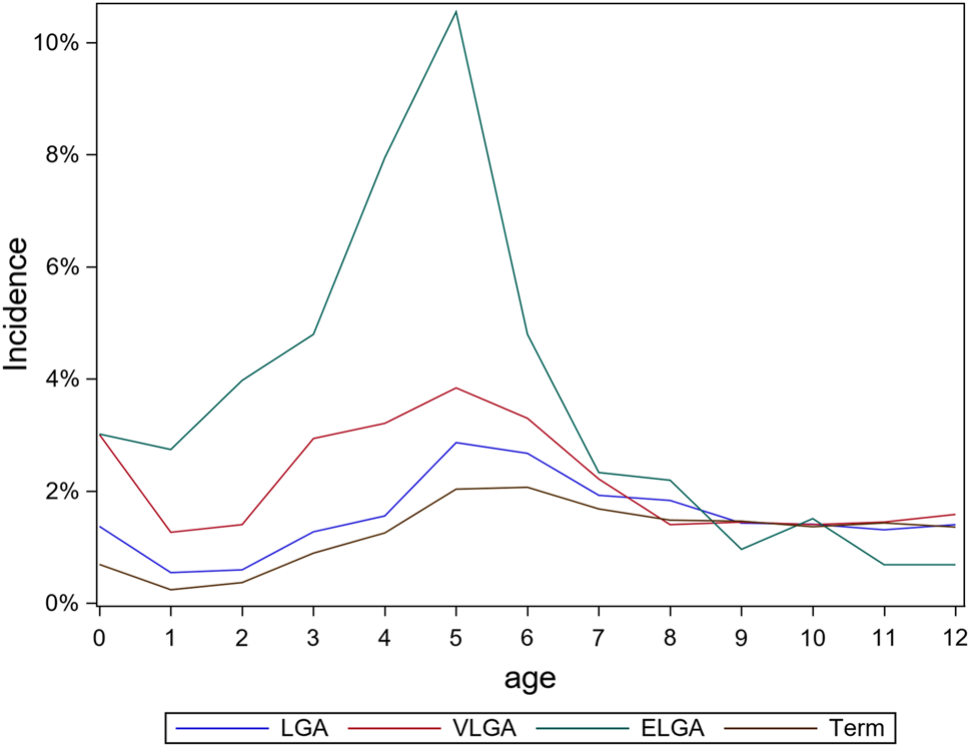
Incidence of any mental, behavioral, or neurodevelopmental disorder at 0 − 12 years in four gestation age groups. *ELGA* extreme low gestation age, *VLGA* very low gestation age, *LGA* low gestation age, and term children

The earlier the child was born, the higher the risk of any, comorbidity and the earlier the diagnosis of mental, behavioral, and neurodevelopmental disorder were (*p* < 0.001); please see Table 2 and Fig. 1. The average number of mental, behavioral, and neurodevelopmental disorders for term children was 0.28 (0.80) and 0.41 (0.96) for PT infants, *p* < 0.0001. Comorbidity was 3–6 times more common in PT children than in term children (Table 2), and the range of different mental health disorders was 0 − 13 in term and 0 − 10 in PT children. The mean age at which any mental, behavioral, or neurodevelopmental disorder was diagnosed for the first time was 4.6 years (2.6) in ELGA, 5.4 years (3.4) in VLGA, 6.4 years (3.3) in LGA, and 7.0 years (3.2) in term children, *p* < 0.0001. The incidence of mental health, behavioral, and neurodevelopmental disorders for these GA subgroups is presented in Fig. 2 and illustrates how ELGA children differ from other studied children, with the higher and earlier incidence peak in early years.

**Table 2:** The rate of comorbidities of mental, behavioral, and neurodevelopmental disorders in children born at extremely low gestation age (ELGA), very low gestation age (VLGA), low gestation age (LGA), and term age

% (*N*)	ELGA (*N* = 796)	VLGA (*N* = 2229)	LGA (*N* = 14,178)	Term (*N* = 309,699)
Two disorders	11.4% (91)	7.5% (168)	4.5% (638)	3.4% (10,619)
Three disorders	6.8% (54)	3.0% (68)	2.0% (293)	1.6% (4951)
Four disorders	4.4% (35)	1.4% (30)	1.1% (150)	0.8% (2461)
Five disorders	1.0% (8)	1.0% (22)	0.5% (77)	0.4% (1129)

Figure 1 displays the prevalence of mental, behavioral, and neurodevelopmental disorders (percent) in children born 22–44 weeks of gestation and followed from birth to 12 years of age.

Extreme low GA (ELGA, ≤ 28 weeks), very low GA (VLGA, 29 − 31), low GA (LGA, 32 − 36) and term (≥ 37) children.

The prevalence of any mental, behavioral, or neurodevelopmental disorders was higher in boys 64.3% (*N* = 34,917) than in girls 35.7% (*N* = 19,353), *p* < 0.0001. Background variables on mothers were chosen for multivariate analysis by preliminary analysis and earlier studies discussed in introduction [9, 10, 19, 52–54]. Some associations between maternal health factors and mental health disorder risk in child were different for PT versus term born children, like earlier birth(s) of mother were risk in term but not in PT children and multiple birth was risk for PT but not for term born children (Table 3). Multiple births were more common in mothers of PT children than in term children, and mothers of PT children were more often first-time mothers than mothers of term children, *p* < 0.001. Mother’s mental, behavioral, and neurodevelopmental disorder associated with disorder of the child (Table 3 and online resource, Table 2), *p* < 0.0001. Mothers of PT children smoked, lived alone, or had mental health disorders more often than mothers of term children (Table 1), *p* < 0.001. In total, 6.3% (*N* = 15,140) of mothers were diagnosed to have any mental health disorder diagnosis during their lifetime until one year before the child was born.

**Table 3:** Multivariate analysis of association between GA and childhood mental, behavioral, and neurodevelopment disorders (unadjusted and adjusted for confounders)

Odds Ratio with 95% Confidence Interval	Unadjusted	When adjusted for confounders
PT vs. term*	1.48 [1.42 − 1.55]	1.37 [1.28 − 1.46]
ELGA versus term	3.99 [3.39 − 4.70]	4.03 [3.08 − 5.26]
VLGA versus term	2.04 [1.84 − 2.26]	1.94 [1.64 − 2.29]
LGA versus term	1.31 [1.25 − 1.38]	1.21 [1.12 − 1.30]
Male versus female gender*		1.94 [1.90 − 1.94]
Maternal mental health disorder or not*		1.99 [1.92 − 2.07]
Smoking versus not*		1.58 [1.54 − 1.62]
Mother living alone versus not*		1.35 [1.31 − 1.40]
Mother out of work life versus not*		1.19 [1.16 − 1.22]
Multiple versus single birth, **separately for		
ELGA^ns^		1.07 [0.71 − 1.61]
VLGA^ns^		0.81 [0.64 − 1.02]
LGA^ns^		0.88 [0.78 − 0.99]
Term^ns^		1.08 [1.00 − 1.18]
Earlier births versus none, ***separately for		
ELGA born^ns^		0.94 [0.67 − 1.32]
VLGA born^ns^		1.09 [0.88 − 1.35]
LGA born^ns^		1.09 [0.99 − 1.20]
Term born*		0.93 [0.91 − 0.95]

Results separately for all subgroups if there were differences in the association between gestational age and confounding variables

*ns* non-significant

*p*-value ≥ 0.05

*p-value < 0.05

**p < 0.05 for preterm and ns. for term children

***p < 0.05 for term and ns for preterm children

## Discussion

This study gathered comprehensive data on all Finnish children born between 2001 and 2006 and followed up to 12 years of age, and it provided new information about the pathway of mental health disorders in general in association with GA and life circumstances before childbirth and pregnancy, here working, relationship, mental health, and smoking history of mother. Notably, the earlier a child was born, the greater the overall risk of any mental, behavioral, or neurodevelopmental disorders; not only that, but the risk of early onset diagnosis of such disorders, as well as multimorbidity in mental, behavioral, and neurodevelopmental disorders, also increased proportionally. Boys had more often diagnoses than girls. The life conditions of the mother were significant for the mental well-being of the child; an experience of any mental health disorder during lifetime before pregnancy in the mother and a habit of smoking during pregnancy were both strongly associated with a risk of a mental health disorder in the child. With less strong impact were living alone and being out of work. This study illustrated that mental health disorders could be recognized in early childhood and emphasize the importance of preventive actions in families with children born early and with psychosocial adversities in family conditions.

Our finding of a strong association between GA and mental, behavioral, and neurodevelopmental disorders is in line with earlier results [2, 19]. Especially, we found that ELGA children stood out from all children with a higher prevalence of any disorder (43% versus 17%) or multiple (≥ 2) disorders (14% versus 3%); this in line with studies on adolescents [55], and adults born PT [4, 5, 9, 43]. Yet, this has been less discussed in childhood mental health studies. It has been speculated that intellectual or psychological development disorders may be mediators for multimorbidity (all diseases) [55]. For examples genetic factors that can cause syndromes, might explain some of multimorbidity. Though, we excluded the most severe and unclear intellectual disabilities and congenital syndromes from the final analysis.

The incidence peak was very early in ELGA children, at under 5 years; in term children, it was around 7 years. This study showed that mental health disorders may be established at young age, and symptoms seems to show early on in children born very PT, which offer the possibility for in-time rehabilitation, preventive intervention, and treatment referral to mitigate potential life-long consequences [18].

Our study results on the mother’s health and life situation association with risks of mental, behavioral, and neurodevelopmental disorders in the offspring follow earlier results [27, 28, 46, 52, 56]. In the present study, we found no differences in the work status of mothers, but mental health disorders were more common in mothers of PT than in term children. Thus, essential differences in mental health outcomes among PT children may relate to the emotional rather than socioeconomic conditions of mothers [57]. Anyhow, the health and life circumstances of mothers may influence their children’s mental development through multicausal pathways [9, 26–28, 56]. In our study, we found that there were more boys than girls, multiple-than-single births, and first-time than multiparous mothers in PT than in term children. Moreover, boys had mental health-related childhood diagnoses more often than girls. Earlier studies reported no or very mild differences between genders [4, 6], but there have been studies with similar results to ours [19, 43]. Based on our results, multiple birth may be a risk for PT children. There is a need for further studies on GA and mental health and related risks with larger samples [44].

This register study offered the possibility to collect data on all children and to study the associations between prenatal-related factors and mental health morbidity in general. Overall, in this study, children born at a GA of 23 weeks had the highest prevalence of mental, behavioral, or neurodevelopmental disorders compared with other children. Because the number of these children is usually small, they are often combined into the ELGA class. In the future, our observations should be tested to verify them in other studies. Unfortunately, as a limitation of our work, we could not study paternal health in association with children’s mental health. Generally, the association of paternal mental, behavioral, and neurodevelopmental disorders with child mental development has been less studied [14, 56]. We assume that this study did not overestimate mental health diagnoses in children, as the most severe disabled children were excluded, and Finnish children are often treated before school age via easy access to preventive services often without diagnostic assessment. Moreover, mental health disorders may be underdiagnosed at younger ages [7]. However, it is plausible that PT children attend developmental follow-up in specialized healthcare settings more often than term controls do [40].

## Conclusion

This study illustrates the importance of structured follow-ups for families with preterm infants, especially those born in extremely early, and possible other stressors to enable prevention and further assessment. Identification of early symptoms of mental, behavioral, or neurodevelopmental disorders, especially in most PT children may be crucial for their later mental health.

### Supplementary Information

Below is the link to the electronic supplementary material.

Supplementary file1 (DOCX 14 kb)
