# Supplementary material for: Burden of mental, behavioral, and neurodevelopmental disorders in the Finnish most preterm children: a national register study
Source: Eur Child Adolesc Psychiatry. 2023 Feb 27;33(2):431–8. doi: 10.1007/s00787-023-02172-1 (PMC10869390; doi:10.1007/s00787-023-02172-1)
Supplement: Supplementary file 1 — Supplementary file1 (DOCX 14 kb) [file 787_2023_2172_MOESM1_ESM.docx]

Online Resource

Table 1 Mental health disorders for all children.

| Number of observations and lifetime prevalence (%) | Children (N = 54,270/326,902) |
| --- | --- |
| F01−09 Mental disorders due to known physiological conditions | 79 (0.02 %) |
| F10−19 Mental and behavioral disorders due to psychoactive substance use | 176 (0.05 %) |
| F20−29 Schizophrenia, schizotypal, delusional, and other non-mood psychotic disorders | 250 (0.08 %) |
| F30−39 Mood [affective] disorders | 3440 (1.05 %) |
| F40−48 Anxiety, dissociative, stress-related, somatoform and other nonpsychotic mental disorders | 7939 (2.43 %) |
| F50−59 Behavioral syndromes associated with physiological disturbances and physical factors | 3578 (1.09 %) |
| F60−69 Disorders of adult personality and behavior | 344 (0.11 %) |
| F70−79 Intellectual disabilities | 1209 (0.37 %) |
| F80−89 Pervasive and specific developmental disorders | 23,801 (7.28 %) |
| F90−98 Behavioral and emotional disorders with onset usually occurring in childhood and adolescence | 31,719 (9.72 %) |
| F99 Unspecified mental disorders | 117 (0.04 %) |

* F72−3 and F79 were excluded

Table 2 Maternal psychosocial risks and mental health disorders in children.

| Number of observations (percentage) | Children with mental health disorder diagnosis (N = 54,270) | Children without mental health disorder diagnosis (N = 272,632) |
| --- | --- | --- |
| Mother living with a partner * | 45,691 (84.2 %) | 242,129 (88.8 %) |
| Mother in work life * | 35,888 (66.1 %) | 193,741 (71.1 %) |
| Mother’s any mental health disorder diagnosis before childbirth * | 6,384 (11.8 %) | 14,947 (5.5 %) |

* There was statistically significant difference between two groups, p < 0.0001.
